# *Echium amoenum* L. Ethanol Extract Protects Retinal Ganglion Cell after Glutamate and Optic Nerve Crush Injury

**DOI:** 10.1155/2022/3631532

**Published:** 2022-09-22

**Authors:** Haibo Li, Ghazaleh Behnammanesh, Zhenkai Wu, Rong Rong, Mengling You, Aman Shah Abdul Majid, Dan Ji

**Affiliations:** ^1^Eye Center of Xiangya Hospital and Hunan Key Laboratory of Ophthalmology, Central South University, Changsha, 410008 Hunan Province, China; ^2^Department of Pharmacology, School of Pharmaceutical Sciences, Universiti Sains Malaysia, Penang, Malaysia; ^3^The First People's Hospital of Changde, Changde City, 415000 Hunan Province, China; ^4^Department of Pharmacology, Quest International University Perak, Ipoh, Malaysia

## Abstract

The development of low-cost and effective natural products for treating neuron degenerative diseases have proven to be safe and potentially effective. *Echium amoenum* L. (Boraginaceae) is an annual herb that grows wildly in Europe and western Asia. The aim of this study was to evaluate the neuroprotective properties of an ethanol extract of *E. amoenum* L. The effects of *E. amoenum* L. extract on oxidative stress were measured in the rat R28 retinal precursor cell line. Furthermore, the protective role of the extract on the glutamate-induced and optic nerve crush (ONC) injury-induced cell death were evaluated *in vitro* and *in vivo*, respectively. Our results showed that the ethanol extract of *E. amoenum* L. prevented the glutamate-induced decrease in cell viability and increase in cell death in R28 cells and suppressed the overproduction of ROS induced by glutamate. Moreover, the extract significantly inhibited microglial activation and optic nerve damage induced by ONC injury in mice. In addition, the mechanism was attributed to the ability of the extract to decrease NF-*κ*B pathway activation and its downstream inflammatory cytokine production. In conclusion, *E. amoenum* L. ethanol extract had a potent neuroprotective effect against glutamate-induced and ONC-induced cell death. This is likely due to its antioxidant and anti-inflammatory properties.

## 1. Introduction

Degenerative diseases of the central nervous system (CNS), injury, and trauma lead to axonal damage, representing major causes of mortality and disability worldwide [1, 2]. Adult CNS neurons in mammals have reduced regenerative potential, leading to permanent impairments and disabilities following injury. Thus, identifying compounds that could be used to protect neurons or help them regenerate after injury is of clinical interest. Currently, the most accepted approach for neuroprotection is oxidative stress reduction using antioxidants, antiapoptotic drugs, anti-inflammatory agents, antiangiogenic agents, and neurotrophic factors [3]. Multiple natural or nutraceutical-based antioxidants have been used as neuroprotective agents to control or slow the development of neurodegenerative ailments, including Alzheimer's disease, amyotrophic lateral sclerosis, ischemic and hemorrhagic stroke, and Parkinson's disease [4–6].

In recent years, the development of low-cost and effective natural products for treating various human ailments has expanded based on large-scale efforts in the screening of crude plant extracts for their biological activities. Commercially, the most important step in developing naturally derived pharmaceuticals is the extraction procedure. The solvent used, yield, and validation assay to assess the pharmacological activities of the extract, availability of raw material, cost, safety of required solvents, and applicability for large-scale production are all important factors to be considered. *E*. *amoenum* L. is an annual herb that grows in most parts of Europe, the Mediterranean region, and northern mountains of Iran. The flowers, stems, roots, and leaves of this plant are used for medicinal purposes. The petals of *E. amoenum* are traditionally brewed or boiled in water, and the flowers and leaves have been used as anti-inflammatory, antioxidant, antibacterial, analgesic, antiviral, anxiolytic, antidepressant, and mood-enhancing agents, and a possible protective effect against cancer has also been proposed [7, 8]. Although *E. amoenum* is extensively used as a medicinal herb in the Middle East, to date, no scientific report has evaluated its potential neuroprotective properties. Therefore, this study assessed the potential neuroprotective features of *E. amoenum* ethanol extract and screened for components that may contribute to such effects.

Optic nerve injury induces the death of 70–75% of retinal ganglion cells (RGCs) within 7 days and 80–90% of cells within 4 weeks, mainly because of apoptosis [9]. RGC death is accompanied by multiple progressive alterations, including glutamate excitotoxicity, ion imbalance, and oxidative stress [10, 11]. The ease of accessibility, coupled with the reproducibility of optic neurodegeneration, makes the optic nerve a highly effective structure for assessing CNS trauma and helps to understand the ensuing traumatic events that activate neuronal apoptosis [12, 13].

Current therapeutic options for neurodegenerative ailments are mainly based on symptomatic relief and not on changing the disease course or progression. Thus, the main aims of this study were to assess whether (a) the potential antioxidant effect of *E. amoenum* ethanol extract could explain its neuroprotective activity and (b) the extract could attenuate the inflammatory reaction associated with optic nerve crush (ONC) injury sustained in the mouse optic nerve. Toward this end, we first analyzed the compounds present in *E. amoenum* ethanol extract and measured their antioxidant activity. We further established mouse and cell models of optic neuropathy to assess the neuroprotective effects of *E. amoenum* ethanol extract *in vitro* and *in vivo*. This study evaluates the potential neuroprotective properties of *E. amoenum* ethanol extracts on the optic nerve at the cellular and molecular levels. These findings could help to promote the clinical treatment of glaucoma optic neuropathy. We also investigated new, effective, and selective natural compounds derived from plants for optic nerve protection to lay an important foundation for the clinical application and large-scale production of natural compounds derived from E. amoenum L.

## 2. Material and Methods

### 2.1. Preparation and Extraction of *E. Amoenum*

Fresh petals of *E. amoenum* were harvested from a farm located 80 km north of Ghazvin, Iran, and authenticated by the Herbarium Unit, School of Biological Sciences, University of Science Malaysia (voucher reference 11525). The extraction was performed as previously described [14]. Briefly, pulverized petals were extracted using the maceration method by adding 800 mL of 98% ethanol to 200 g of plant material at room temperature (RT; 25 ± 2°C) for 48 h with intermittent shaking. The resulting extract was filtered and concentrated under reduced pressure using a rotary evaporator (BUCHI, Germany) at 35°C and lyophilized in a freeze dryer (Labconco, USA). The stock solution was stored at 2–8°C until use.

### 2.2. Total Phenolic Content Assessment

The total phenolic content of the extract was obtained as described previously [15], with gallic acid as the standard. One milliliter of gallic acid (1–30 *μ*g/mL) in distilled water was added to 0.5 mL of Folin-Ciocalteu reagent (Merck, Darmstadt, Germany), incubated for 4 min at RT, and mixed with 20% Na_2_CO_3_ (1 mL) and distilled water (6 mL). The mixture was incubated in the dark for 2 h at RT, and the absorbance at 765 nm was measured using a Tecan microplate reader. The *E. amoenum* ethanol extract stock solution (1 mg/mL) was prepared using methanol. To determine the total phenolic content of the extract, Folin-Ciocalteu reagent, Na_2_CO_3_, test samples, and distilled water were added to a test tube, followed by the same procedures described above for gallic acid. Folin-Ciocalteu reagent is an acidic solution with a yellow color, which changes to blue as a result of molybdenum-tungsten blue complex production through the electron transfer mechanism. The data are presented in units of *μ*g gallic acid equivalents (GAE)/g extract.

### 2.3. Total Flavonoid Level Assessment

A colorimetric assay was employed to determine the total flavonoid levels in the extract, with quercetin as a reference. Stock solutions of 2 mg/mL *E. amoenum* ethanol extract and 1 mg/mL quercetin were prepared in methanol. For the assay, 1 mL of standard solution (1–30 *μ*g/mL in distilled water) or extract was added to 4 mL of distilled water and 0.3 mL of 5% sodium nitrite. The sample was incubated for 5 min at RT, followed by the addition of 0.3 mL of 10% (w/v) aluminum chloride. Subsequently, 2 mL of 1 M NaOH was added with distilled water to reach a volume of 10 mL. The optical density (OD) was measured at 510 nm using a Tecan microplate reader. For the blank assay, sodium nitrite and aluminum chloride were replaced with water.

### 2.4. Gas Chromatography-Mass Spectroscopy (GC-MS)

GC-MS was used to detect the volatile constituents of the extract [16] on an Agilent GC-MS system (6890 N/5973I) equipped with a single quadruple detector and an HP-5 MS capillary column (0.25 mm × 30 m × 0.25 *μ*m). The injection volume used was 1 *μ*L. The *E. amoenum* components were confirmed using the NIST library identified with standards (retention time and mass spectra).

### 2.5. Radical Scavenging Activity

#### 2.5.1. 2,2-Azinobis (3-Ethyl-Benzothiazoline-6-Sulfonic Acid) (ABTS) Assay

Radical scavenging activity was first tested using the ABTS assay, with some modifications regarding sample volumes and the ABTS solution. This assay assesses the capacity of compounds to scavenge the stable ABTS radical cation (ABTS^+^). Fresh ABTS radicals were obtained by mixing 2.45 mM potassium persulfate and 7 mM ABTS aqueous solutions (2.5 mL each), and the mixture was maintained in the dark for 16–20 h at RT. This solution was adjusted to an OD at 734 nm of 0.70 ± 0.02. A stock solution of the test extract and standard (ascorbic acid) at 1 mg/mL was diluted in methanol to provide a series of working solutions of 1, 5, 10, 20, and 30 *μ*g/mL. Final reaction mixtures consisting of 0.9 mL of ABTS and 0.1 mL of test sample solutions at various concentrations were thoroughly mixed and incubated for 6 min at RT. For blank samples, methanol (0.9 mL) was added to the sample solution at different concentrations (0.1 mL). ABTS solution (0.9 mL) was mixed with methanol (0.1 mL) as a negative control. The OD value was measured at 518 nm using a Tecan microplate reader. The percentage scavenging antioxidant activity was calculated as ([*A*_0_–(*A*_1_–*A*_2_)]/*A*_0_) × 100, where *A*_0_, *A*_1_, and *A*_2_ are the OD values of the control sample, test extract/standard, and corresponding blank, respectively.

#### 2.5.2. 2,2-Diphenyl-1-Picrylhydrazyl (DPPH) Assay

The DPPH assay was performed as previously described [17]. The *E. amoenum* ethanol extract was diluted in methanol to concentrations of 1, 5, 10, 20, and 30 *μ*g/mL. The samples (2.5 mL) in methanol:water (1 : 1) were then mixed with 1 mL of 0.3 mM DPPH in methanol. For the blank samples, methanol (1 mL) was mixed with various extract concentrations. Rutin concentrations of 1, 5, 10, 20, and 30 *μ*g/mL were used as the reference standard. As a negative control, 2.5 mL methanol and 1 mL of 0.3 mM DPPH were mixed. The mixtures were incubated at RT for 30 min to allow stable DPPH radicals to be reduced to diphenyl-picrylhydrazine by antioxidants in various solutions. OD values were obtained at 517 nm, and the background value was subtracted. Data are presented as half-maximal inhibitory concentration (IC_50_) ± standard error of the mean (SEM), representing the concentration with a scavenging activity of 50%. The percentage of free radical-scavenging activity was calculated as ([*A*_0_–(*A*_1_–*A*_2_)]/*A*_0_) × 100, where *A*_0_, *A*_1_, and *A*_2_ are the OD values of the control sample, test extract/standard, and corresponding blank, respectively.

#### 2.5.3. *β*-Carotene Bleaching Assay

The *β*-carotene bleaching assay was performed as previously described [18]. First, 1 mL of *β*-carotene in chloroform (0.2 mg/mL) was added to linoleic acid (20 mg) and 200 mg Tween 40. After the chloroform was removed using a rotary evaporator, distilled water (50 mL) was used for emulsification. Emulsion aliquots were mixed with 0.2 mL of the *E. amoenum* ethanol extract at 1, 5, 10, 20, and 30 *μ*g/mL. Butylated hydroxytoluene (BHT; 0.5, 1, 5, 10, and 20 *μ*g/mL) was used as the reference standard. The initial OD was obtained at 470 nm on a Tecan microplate reader immediately after emulsion addition. The samples were incubated at 50°C in a water bath, and OD values were obtained every 20 min until a color change in the control samples was observed. Antioxidant activity was calculated as (final *β* − carotene amount/initial *β* − carotene amount) × 100. The IC_50_ value of the extract was obtained from the curve of antioxidant activity percentage versus the extract level.

### 2.6. Cell Viability and Cell Death Assays in R28 Cells

Rat retinal precursor R28 cells were used as an *in vitro* model owing to their similar characteristics to retinal neurons (Kerafast, Boston, MA, USA). Cell viability was measured using the Cell Counting Kit-8 (CCK-8; NCM Biotech, China). Hoechst 33342-propidium iodide dual staining (Beyotime, Shanghai, China) was used to detect cell death, according to the manufacturer's instructions.

### 2.7. Reactive Oxygen Species (ROS) Production

R28 cells were treated with the ethanol extract of *E. amoenum* (50 or 100 *μ*g/mL) and 10 mM glutamate for 12 h, digested with collagenase IV (Gibco, Waltham, MA, USA), and then suspended in medium containing 10 *μ*M of the fluorescent probe 2′-7′-dichlorofluorescin diacetate (Sigma-Aldrich, St. Louis, MO, USA). After 30 min of incubation at 37°C in the dark, the cells were centrifuged at 1000 g, resuspended in fresh medium, and subjected to fluorescence activated cell sorting analysis. The mean fluorescent intensity was compared between the treated groups and control group using FlowJo 7.6 software (Tree Star Inc., Ashland, USA). The experiments were repeated three times.

### 2.8. Animals

BALB/c mice were used in this study. All animal experiments were conducted in accordance with the Animal Ethics Guidelines of Xiangya Hospital, Central South University (permit number: 202108022). During the experimental period, all mice were maintained in standard cages in an animal room under normal environmental conditions. All surgery was performed under anesthesia, and all efforts were made to minimize suffering. Twelve male mice (8–12 weeks old) were randomly divided into four groups: the normal control and negative control groups and two groups of test animals (*n* = 3 per group). The two control groups were fed a normal diet. For the two test groups, the mice were orally administered 200 mg/kg and 400 mg/kg of ethanol extract once daily for 10 days. Subsequently, ONC was performed as described below. In this study, no participants have involved in this basic research, so there is no need to provide informed consent.

### 2.9. ONC Model

Mice were anesthetized by intraperitoneal injection of ketamine (90 mg/kg) and xylazine (10 mg/kg). Unilateral ONC injury was induced in the negative control and test groups, as described previously [19]. The optic nerve of the left eye was pressed 0.5 mm posterior from the globe for 10s using Jeweler's forceps without damaging the ocular blood vessels. The contralateral eye was used as the uncrushed control.

### 2.10. Immunocytochemistry and Immunostaining of Flat-Mounted Retinas

For the retina immunohistochemistry analysis, the eyes were dissected and fixed in 4% paraformaldehyde at RT for 2 h and dehydrated in 20% sucrose before embedding in O.C.T. mounting medium. Frozen sections (16 *μ*m) were then cut using a cryostat microtome (CM1860; Leica, Nussloch, Germany). Immunohistochemistry was performed using anti-Iba1 rabbit antibody (Abcam, ab178847, 1/300). The nuclei were stained with 4′,6-diamidino-2-phenylindole (DAPI) for 2 min. Slides were examined using a fluorescence microscope (Leica, Wetzlar, Germany). Iba1-positive cells were quantitatively analyzed using ImageJ software.

For immunostaining of flat-mounted retinas, the eyes of mice in the ONC group with or without *E. amoenum* ethanol extract treatment were enucleated and fixed in 4% paraformaldehyde for 1 h at RT. The retinas were peeled off, prepared as flattened whole mounts, and permeabilized in 0.5% Triton X-100 in phosphate-buffered saline (PBS) for 15 min at RT. The flat-mounted retinas were then washed with PBS and incubated overnight at 4°C with anti-RBPMS rabbit polyclonal antibody (Thermo Fisher Scientific, PA5-31231, 1/300) and anti-Tuj1 mouse monoclonal antibody (Millipore, MAB1637, 1/300). The nuclei were stained with DAPI for 2 min, and flat-mounted retinas were examined using a fluorescence microscope (Leica). Each image was obtained from eight 20× fields around the peripheral retina (two in each quadrant) for each whole-mounted retina. The number of RBPMS-positive cells, as a marker of RGCs, in each image was quantified using ImageJ software as described previously [20].

### 2.11. Immunoblotting

Immunoblotting was performed as described previously [21], with the following primary antibodies: mouse anti-Neurofilament light chain (NF-L) (Cell Signaling Technology, 2837S, 1/1000), antiphospho-NF-*κ*B p65 (Ser536) rabbit antibody (Cell Signaling Technology, 3033S, 1/1000), anti-NF-*κ*B p65 rabbit antibody (Cell Signaling Technology, 8242S, 1/1000), and mouse anti-actin (Cell Signaling Technology, 3700S, 1/5000). Mouse retinas of each group were isolated and sonicated in 150 *μ*L of homogenization buffer (20 mM Tris/HCl, pH 7.4, containing 2 mM EDTA, 0.5 mM EGTA, 1% sodium dodecyl sulfate, 0.1 mM phenylmethylsulfonyl fluoride, 50 *μ*g/mL aprotinin, 50 *μ*g/mL leupeptin, and 50 *μ*g/mL pepstatin A). An equal volume of sample buffer (62.5 mM Tris/HCl, pH 7.4, containing 4% sodium dodecyl sulfate, 10% glycerol, 10% mercaptoethanol, and 0.002% bromophenol blue) was immediately added, and samples were boiled for 2–3 min. The protein content was determined using a bicinchoninic acid protein kit (Sigma-Aldrich). Equal amounts of protein were separated by electrophoresis on 10% polyacrylamide gels containing 0.1% sodium dodecyl sulfate. Proteins were transferred to nitrocellulose membranes and the blots were incubated for 2 h at room temperature with primary antibodies. Detection was performed using the appropriate horseradish-conjugated secondary antibodies for 1 h at RT. Bands were visualized using an enhanced chemiluminescence solution and quantified using ImageJ software (Wayne Rasband, National Institutes of Health, Bethesda, MD, USA).

### 2.12. Quantitative Real-Time Polymerase Chain Reaction (PCR)

Total RNA of mouse retinas was obtained using RNeasy® Kit (Qiagen), according to the manufacturer's instructions, and quantified on a NanoDrop 1000 spectrophotometer (Thermo Scientific, Waltham, MA, USA). Reverse transcription was performed using Oligo dT and Superscript III reverse transcriptase (Invitrogen, 11732020). Primers were manufactured by Sangon Biotech (Shanghai, China), and the following primer sequences were used: *Il6* (forward 5′-GTGGCTAAGGACCAAGACCA-3′ and reverse 5′-ACCACAGTGAGGAATGTCCA-3′), *Il1b*(forward5′-GCAACGGGAAGATTCTGAAG-3′ and reverse 5′-TGACAAACTTCTGCCTGACG-3′), iNOS (forward 5′-ACGAGACGGATAGGCAGAGA-3′ and reverse 5′-CACATGCAAGGAAGGGAACT-3′), and beta-actin (forward 5′-CACGATGGAGGGGCCGGACTCATC′ and reverse 5′-TAAAGACCTCTATGCCAACACAGT-3′).

Real-time PCR was performed with SYBR Green I Master mix (Applied Biosystems) in triplicate reactions (20 *μ*L) containing 1 *μ*L of cDNA template with 500 nM primers, at 95°C (10 min), followed by 40 cycles of 95°C (15 s) and 60°C (1 min), and finally 78°C for 20 s. Melting curve generation was performed using Dissociation Curves software (Applied Biosystems). The 2^-*ΔΔ*CT^ method was used for the data analysis.

### 2.13. Statistical Analysis

Statistical analyses were performed using GraphPad Prism software (version 8.0). One-way analysis of variance with Tukey's multiple comparison test was used for normally distributed variables or the Kruskal-Wallis test was used for variables with a skewed distribution. Data are presented as the mean percentage of control ± SEM. Statistical significance was set at *P* < 0.05.

## 3. Results

### 3.1. Antioxidant Activities of the *E. amoenum* Ethanol Extract

**Figure 1:**
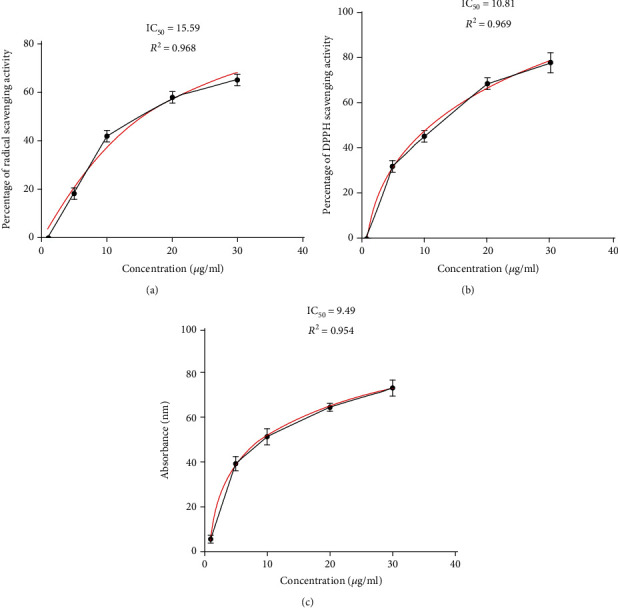
Antioxidant activities of *E. amoenum* ethanol extract. The free radical-scavenging activities of *E. amoenum* ethanol extract were measured using ABTS **(**a**)**, DPPH (b), and *β*-carotene bleaching (c) assays. Data are shown as the mean ± SEM from three experiments.

The total phenolic content of the dry *E. amoenum* ethanol extract was 1390 ± 22 *μ*g GAE/g. The total flavonoid content of the dry extract was 9360 ± 83 *μ*g QE/g.  Figure 1(a) shows the free radical-scavenging activity of the *E. amoenum* ethanol extract in the ABTS assay, with an IC_50_ value of 15.59 *μ*g/mL. Ascorbic acid, the reference antioxidant, displayed an IC_50_ of 4.97 ± 0.06 *μ*g/mL.  Figure 1(b) shows that the extract had a strong antioxidant effect, as indicated by the low IC_50_ of 10.81 *μ*g/mL; the reference rutin had an IC_50_ of 3.23 ± 0.02 *μ*g/mL. These findings indicated that the extract potently scavenged the stable free radical DPPH. The *β*-carotene bleaching effects of the extract are shown in Figure 1(c). The IC_50_ of the standard (BHT) was 0.89 ± 0.04 *μ*g/mL. The low IC_50_ of 9.49 *μ*g/mL of the extract indicated its high *β*-carotene bleaching activity. GC-MS analysis revealed the presence of 13 phytochemical constituents in the extract (Table 1), with linolenic acid, campesterol, and *γ*-sitosterol as the major components.

### 3.2. Protective Effects of the E. amoenum Ethanol Extract on Glutamate-Induced R28 Cell Death

**Figure 2:**
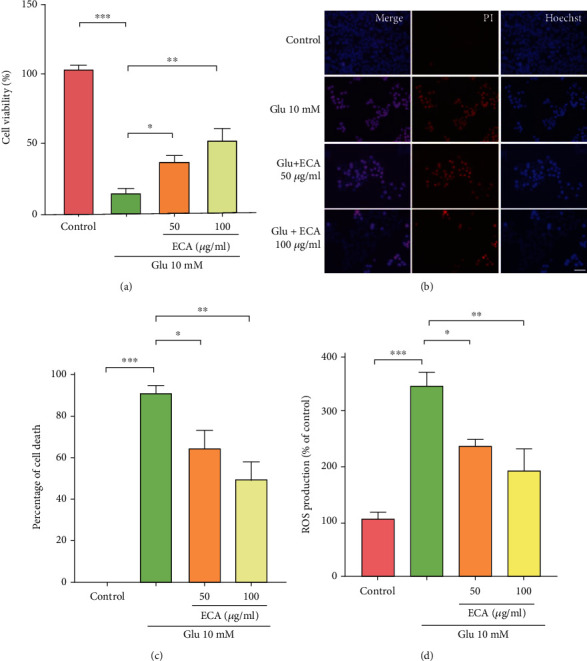
Protective effects of the *E. amoenum* ethanol extract on glutamate-induced R28 cell death. **(**a) Effects of 50 and 100 *μ*g/mL *E. amoenum* ethanol extract on the viability of R28 cells treated with 10 mM glutamate for 24 h, as measured by the CCK-8 assay. (b) Effects of 50 and 100 *μ*g/mL *E. amoenum* ethanol extract on the death of R28 cells treated with 10 mM glutamate for 24 h. (c) Quantification of the percentage of propidium iodide (PI)-positive cells. (d) Fluorescence-activated cell sorting to detect ROS production in glutamate-treated R28 cells. Scale bar = 20 *μ*m. Data are mean ± SEM (*n* = 6); ^∗^*P* < 0.05, ^∗∗^*P* < 0.01, and ^∗∗∗^*P* < 0.001 versus the negative control group. ECA: *E. amoenum* ethanol extract; Glu: glutamate; ROS: reactive oxygen species.

R28 cells treated with 10 mM glutamate for 24 h showed significantly reduced viability (15.3% compared with the control). Moreover, treatment with 50 and 100 *μ*g/mL *E. amoenum* ethanol extract increased cell viability by approximately 21.6% and 36.6%, respectively (Figure 2(a)). Further support for the neuroprotective effect of *E. amoenum* ethanol extract was obtained by propidium iodide dual staining (Figures 2(b) and 2(c)). Administration of 10 mM glutamate resulted in massive cell death. More specifically, the percentage of PI-positive cells in the glutamate-treated group for 24 h was 90.4% ± 3.6. Treatment with 50 and 100 *μ*g/mL *E. amoenum* ethanol extracts reversed these glutamate-induced effects, with higher efficacy found for the higher dose (the percentage of PI-positive cells decreased to 63.9% ± 8.4 and 48.9% ± 8.7, respectively). As shown in Figure 2(d), R28 cells treated with glutamate showed a massive increase in ROS production, which was significantly decreased with treatment of *E. amoenum* ethanol extract (50 and 100 *μ*g/mL). Collectively, these results demonstrated that *E. amoenum* ethanol extract protects against glutamate-induced oxidative stress and cell death.

### 3.3. Neuroprotective Effects of *E. amoenum* Ethanol Extract in ONC Mouse Model

**Figure 3:**
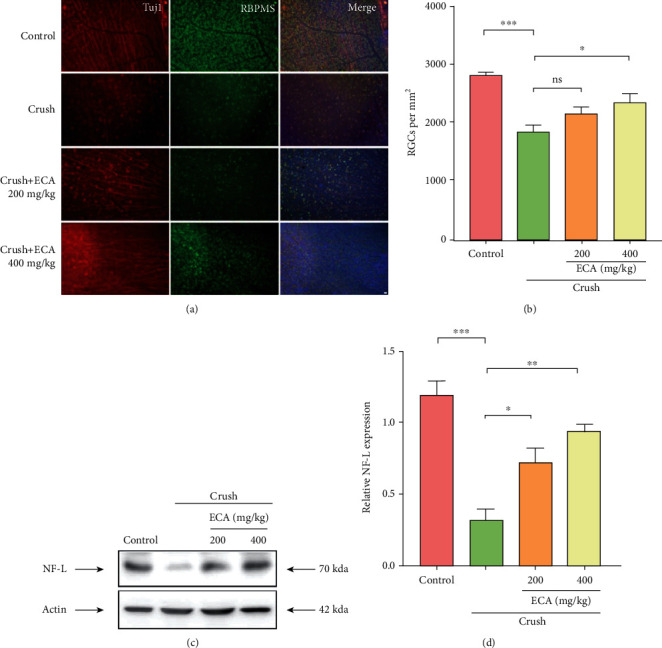
Neuroprotective effects of *E. amoenum* ethanol extract in the optic nerve crush (ONC) mouse model. (a) Retinas from different groups were harvested on day 7 after ONC, and flat-mounted retinas were subjected to immunostaining with RBPMS and Tuj1 antibodies. (b) Quantification of the number of RGCs in 1-mm^2^ retina sections. (c) Expression of the optic nerve protein NF-L determined by immunoblotting. (d) Quantification of the NF-L levels. Relative protein levels were calculated using ImageJ software. Results were obtained from three independent experiments. Scale bar = 50 *μ*m. Data are mean ± SEM; ^∗^*P* < 0.05, ^∗∗^*P* < 0.01, and ^∗∗∗^*P* < 0.001. RGC: retinal ganglion cell; ECA: *E. amoenum* ethanol extract.

After image acquisition and data recording of flat-mounted retinas, the number of RBPMS-positive cells (representing RGCs) was calculated in a 1-mm^2^ section. ONC induced a significant decrease in RGCs, whereas the number of RGCs in animals administered 200 mg/kg and 400 mg/kg of *E. amoenum* ethanol extract was markedly increased compared with that in the ONC group, suggesting attenuation of RGC injury in response to ONC (Figures 3(a) and 3(b)). Neurofilament light chain (NF-L) is a neuronal intermediate filament in optic nerve axons that has been used as an indicator of optic nerve injury. ONC also reduced the NF-L protein levels, whereas administration of *E. amoenum* ethanol extract (200 or 400 mg/kg) significantly alleviated this effect (Figure 3(c)). Specifically, the decrease rates for NF-L protein after ONC in the negative control, 200 mg/kg *E. amoenum* ethanol extract, and 400 mg/kg *E. amoenum* ethanol extract groups were 87.6%, 47.2%, and 28.6%, respectively (Figure 3(d)). Taken together, these findings demonstrate that *E. amoenum* ethanol extract has a neuroprotective effect in an *in vivo* ONC model.

### 3.4. *E. amoenum* Ethanol Extract Suppressed the ONC-Induced Inflammation Response

**Figure 4:**
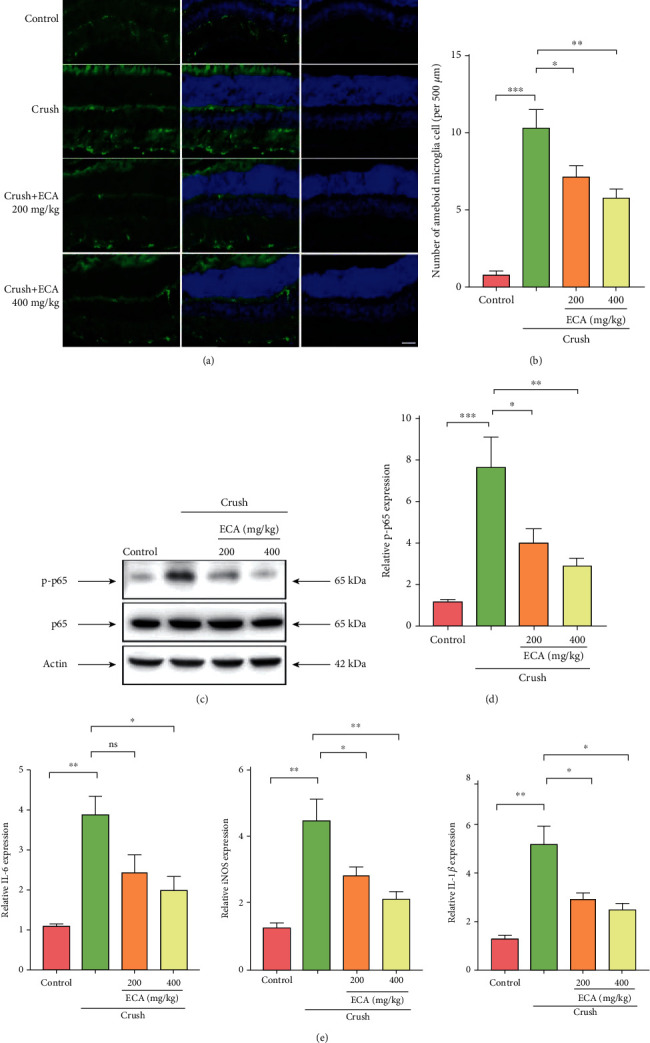
*E. amoenum* ethanol extract suppressed the inflammation response triggered by optic nerve crush (ONC) injury. **(**a**)** Retinas from different groups were harvested on day 7 after ONC and subjected to frozen-section immunostaining with Iba1. (b) Quantification of microglia in the retina. (c) Expression of p-p65 in different groups determined by immunoblotting. (d) Quantification of the p-p65 levels in each group. (e) Retinas from different groups were harvested 7 days after ONC and subjected to real-time PCR analysis to determine the relative mRNA levels of IL-6, iNOS, and IL-1*β*. Data are shown as the mean ± SEM (*n* = 3); ^∗^*P* < 0.05, ^∗∗^*P* < 0.01, and ^∗∗∗^*P* < 0.001.

To determine the anti-inflammatory effects of *E. amoenum* ethanol extract in the ONC mouse model, Iba1-positive cells were detected in the frozen sections of each group as a marker of ameboid microglia cells. The *E. amoenum* ethanol extract group showed a reduced number of activated microglia cells (Figures 4(a) and 4(b)). To explore the mechanism underlying the anti-inflammatory effect of *E. amoenum* ethanol extract, phospho-NF-*κ*B p65 levels in ONC mice treated with or without *E. amoenum* ethanol extract were compared by immunoblotting. Interestingly, phospho-p65 levels in the 200 and 400 mg/kg groups were significantly decreased compared with the control values (Figures 4(c) and 4(d)). As the downstream targets of NF-*κ*B activation, the elevated mRNA levels of proinflammatory cytokines such as IL-6, iNOS, and IL-1*β* triggered by glutamate were significantly decreased upon *E. amoenum* ethanol extract treatment (Figure 4(e)). Taken together, these results suggest that inhibition of the NF-*κ*B pathway by *E. amoenum* ethanol extract blocks ONC-induced microglial activation and proinflammatory cytokine release.

## 4. Discussion

The complex process of neurodegeneration can be initiated as a result of serious acute traumatic injury or chronic intermittent and progressive damage by ischemic and hypoxic conditions associated with oxidative stress, e.g., in Alzheimer's, Parkinson's, and Huntington's diseases, as well as in optic neuropathies. Neuroprotection refers to any therapeutic strategy for the prevention, delay, or reversal of neuron damage or death associated with a given pathology [1, 2, 4, 22].

*E. amoenum* extract has shown anti-inflammatory, anti-oxidative effects and neuroprotective activity in traditional medicine. Recently, a study reported that *E. amoenum* extract could modulate the inflammatory modes of the macrophages through the inhibition of iNOS and COX2 enzymes as well as through cytokines expression [23], It has also been suggested the pharmacological effects of *E. amoenum* extract in treating patients with depression, generalized anxiety disorder, or Alzheimer's disease [24, 25]. However, its role in alleviating or protecting against CNS injury and the underlying mechanisms remain unclear. Here, we demonstrated that the simple crude ethanol extract of *E. amoenum* showed antioxidant capacity in the ABTS and DPPH tests, as well as in the *β*-carotene bleaching assay. These results confirmed that the extract is a potent antioxidant, as supported by previous studies [7, 26]. The extract also showed free radical-scavenging and neuroinflammatory mediator effects in an ONC mouse model. Thus, the current study is the first to assess the neuroprotective features of *E. amoenum* and to highlight the potential application of this herb as a model of neuronal injury in a mouse model of retinal neurodegeneration.

Further phytochemical screening of the extract revealed various constituents such as linolenic acid, *γ*-sitosterol, and campesterol, which together may exert synergistic effects leading to the observed neuroprotective activity. These three compounds were also found in a *Rhus coriaria* extract, which showed beneficial effects in a mouse model of ischemic optic neuropathy [27], supporting the results of the present study. Another study showed the beneficial effects of these compounds in *Drosophila* models of neurodegenerative diseases [28]. In addition, since linolenic acid, *γ*-sitosterol, and campesterol were found at high levels in the extract, they could be used as markers for the standardization of *E. amoenum* ethanol extract and its development as a functional neuroprotective agent.

Microglia undergo different phenotypic changes in different disease states, and several studies have found that the activation of microglia has both protective and damaging effects. When the inflammatory state persists for a long time, the reaction products of microglia may cause damage to the surrounding tissues and even affect healthy cells, especially neurons. Many studies have shown that microglia-mediated inflammation is involved in RGC death, and thus inhibiting the immune response of microglia has a protective effect on RGCs [29, 30]. A similar phenomenon was observed in the present study as *E. amoenum* ethanol extract inhibited microglial activation and cytokine production. NF-*κ*B is involved in many signaling pathways, including inflammation, development, cell growth, and apoptosis [31]. Glutamate-associated apoptosis and NF-*κ*B expression are correlated in vascular endothelial cells, cardiomyocytes, and RGCs [31, 32]. Therefore, NF-*κ*B is considered a potential drug target for novel treatments that reduce inflammation and apoptosis [33]. Consistent with these previous studies, the present study showed that ONC upregulated the expression of molecules associated with the NF-*κ*B pathway, whereas *E. amoenum* ethanol extract significantly downregulated NF-*κ*B expression and its downstream cytokine production. Of course, there is a strong possibility that other pathways might be involved in the observed effects, and thus additional studies are needed to determine the exact mechanisms of *E. amoenum* ethanol extract.

In summary, *E. amoenum* ethanol extract demonstrated potent neuroprotective effects in an *in vivo* model of retinal neurodegeneration, likely because of its antioxidant and anti-inflammatory properties. Accordingly, the present findings support the premise that *E. amoenum* is a bioactive herb that should be further evaluated as an adjunctive neuroprotective agent for preventing neurodegenerative ailments such as glaucoma and neurodegenerative diseases.

## Figures and Tables

**Table 1:** Chemical composition of the ethanol extract of *E. amoenum* determined using GC-MS.

Peak no.	R. time	Compound	Ref	Quality	Peak height	% area	RI
1	10.29	Palmitic acid, methyl ester	100707	99	703156	1.56	
2	10.62	n-Hexadecanoic acid	92228	96	561497	3.78	419.4
3	11.11	9,12-Octadecadienoic acid, methyl ester	114374	99	1667189	2.41	1394.8
4	11.15	Tricosane	113306	99	1123829	1.77	1479.7
5	11.22	Heptadecanoic acid 16-methyl-, methyl ester (methyl isostearate)	116689	98	562581	0.85	1627.2
6	11.43	9,12-Octadecadienoic acid	106289	99	701410	1.70	2026.6
7	11.95	Campesterol	107653	96	6852862	9.35	2300.1
8	12.36	Tetracosane	136482	95	782762	1.19	2399.9
9	13.76	Linolenic acid, (methyl ester)	130016	97	6070549	11.84	2699.9
10	13.24	Docosane	123096	95	471347	1.05	2600.1
11	15.06	Gamma-sitosterol	158131	99	3341735	9.14	2948.6
12	19.38	Eicosane	156589	91	445259	5.14	3771.1
13	20.88	Nonacosane	159285	99	604971	5.57	4056.9

## Data Availability

The data used to support the findings of this study are available from the corresponding author upon request.

## Abbreviations

CNS: Central nervous system
CCK-8: Cell Counting Kit-8
DAPI: 4′,6-diamidino-2-phenylindole
*E. Amoenum*: *Echium amoenum* L
ECA: *E. amoenum* ethanol extract
Glu: Glutamate
ONC: Optic nerve crush
ROS: Reactive oxygen species.
